# Photonic-Assisted Scheme for Simultaneous Self-Interference Cancellation, Fiber Dispersion Immunity, and High-Efficiency Harmonic Down-Conversion

**DOI:** 10.3390/mi14020339

**Published:** 2023-01-28

**Authors:** He Li, Zihang Zhu, Congrui Gao, Guodong Wang, Tao Zhou, Xuan Li, Qingqing Meng, Yixiao Zhou, Shanghong Zhao

**Affiliations:** 1 College of Information and Navigation, Air Force Engineering University, Xi’an 710077, China; 2 Key Laboratory of Electronic Information Control, Southwest China Research Institute of Electronic Equipment, Chengdu 610036, China

**Keywords:** self-interference cancellation, fiber-dispersion-induced power fading, harmonic down-conversion

## Abstract

A photonic approach to the cancellation of self-interference in the optical domain with fiber dispersion immunity and harmonic frequency down-conversion function is proposed based on an integrated, dual-parallel, dual-drive Mach–Zehnder modulator (DP-DMZM). A dual-drive Mach–Zehnder modulator (DMZM) is used as an optical interference canceller, which cancels the self-interference from the impaired signal before fiber transmission to avoid the effect of fiber transmission on the cancellation performance. Another DMZM is used to provide carrier-suppressed, local-oscillation (LO)-modulated, high-order double optical sidebands for harmonic frequency down-conversion to release the strict demand for high-frequency LO sources. By regulating the DC bias of the main modulator, the signal of interest (SOI) can be down-converted to the intermediated frequency (IF) band after photoelectric conversion with improved frequency-conversion efficiency, immunity to the fiber-dispersion-induced power-fading (DIPF) effect, and effective signal recovery. Theoretical analyses and simulation results show that the desired SOI in the X and K bands with a bandwidth of 500 MHz and different modulation formats can be down-converted to the IF frequency. The self-interference noise with the 2 GHz bandwidth is canceled, and successful signal recovery is achieved after a 10 km fiber transmission. The recovery performance of down-converted signals and the self-interference cancellation depth under different interference-to-signal ratios (ISRs) is also investigated. In addition, the compensation performance of DIPF is verified, and a 6 dB improvement in frequency conversion gain is obtained compared with previous work. The proposed scheme is compact, cost-effective, and thus superior in wideband self-interference cancellation, long-range signal transmission, and effective recovery of weak desired signals.

## 1. Introduction

In recent years, industries such as mobile communication, artificial intelligence, and Internet of Things (IoT) are experiencing exponential growth as the number of wireless communication users continues to increase dramatically. This results in the ever-increasing demand for higher data rates and a larger communication capacity [1]. This demand impels us continuously to study techniques that can improve communication traffic and speed while making reasonable and full use of the limited spectrum of resources [2]. The in-band full-duplex (IBFD) is an emerging technique that provides a promising solution to double the spectrum utilization efficiency and data transmission rate by employing the same frequency in the downlink and uplink [3,4]. Furthermore, the integration of Radio-over-fiber (RoF) systems and IBFD technology has generated new IBFD RoF systems, which have the advantages of long-distance transmission, large communication capacity, simplified base station (BS) structure, immunity to electromagnetic interference, enhanced safety, and increased signal data rate [5]. It provides an effective and low-cost solution to future high-speed, large bandwidth, increased capacity, and large-scale cellular wireless networks for both mobile and fixed users [6,7,8]. However, IBFD RoF systems mainly face three long-standing problems. First, due to the in-band operation and co-site located transmitting and receiving antennas, the high-power transmitting signals interfere with the weak received signal of interest (SOI) [9], which cannot be eliminated simply by using electrical filters. Second, the dispersion of the single-mode fiber (SMF) leads to a periodical fiber-dispersion-induced power-fading (DIPF) effect limiting its application for high-frequency radio frequency (RF) carrier and long-distance fiber transmission [10]. Third, the SOI signals in higher-frequency bands require down-conversion so as to alleviate the strict demand for a high sampling ratio analog-to-digital converter [11].

Previously, considerable efforts have been devoted to solving the above three problems. To date, the reported works based on microwave photonic technology have shown the potential for deep self-interference cancellation (SIC) over a wide frequency band [12,13,14], good compatibility with fiber transmission [15,16,17], and high-performance frequency conversion [18,19,20]. All three kinds of schemes feature broad instantaneous bandwidth, high operation frequency, electromagnetic interference immunes, and compact configuration compared with their electrical counterparts [21,22]. However, the frequency conversion function in the aforementioned schemes is based on the fundamental frequency tones, which requires a high-frequency local oscillation (LO) source for received signals in higher-frequency bands. Therefore, a harmonic down-conversion (HDC) system is proposed to simultaneously realize SIC, DIPF elimination, and harmonic down-conversion for an IBFD system [23]. The HDC operation in this scheme releases the requirement for a high-frequency LO source through RF signal single sideband (SSB) modulation over a wide bandwidth. Nevertheless, when beating with one LO-modulated high-order single optical sideband generated from SSB modulation, the frequency conversion efficiency (CE) of down-converted signals is low, which is not conducive to the effective recovery of received weak signals.

In this paper, a photonic-assisted scheme for simultaneous ultrawideband SIC, high-speed SOI recovery, and fiber dispersion immunity—which also has the capability of harmonic down-conversion with improved conversion efficiency—is proposed. The self-interference is directly canceled before fiber transmission in the optical domain, which enables the self-interference cancellation performance to be immune to the influence caused by fiber dispersion. By beating the LO-modulated high-order double optical sidebands, the SOI signals in higher-frequency bands can be down-converted to the intermediate frequency (IF) to realize harmonic frequency down-conversion, which releases the strict demand for high-frequency LO sources. Furthermore, the DIPF compensation and frequency conversion efficiency improvement are implemented by virtue of the DC-bias-induced phase control mechanism. The simulation results show that the proposed scheme can perform simultaneous wideband SIC and harmonic frequency conversion in different frequency bands. The recovery performance of SOI with different modulation formats and under different interference-to-signal ratios (ISRs) are investigated, showing an effective signal recovery. Furthermore, DIPF compensation performance is verified via frequency conversion improvement. The proposed scheme has a compact configuration and is suitable for distributed IBFD RoF systems, which is conducive to the improvement in signal transmission performance and the effective recovery of weak desired signals.

## 2. Principle

**Figure 1:**
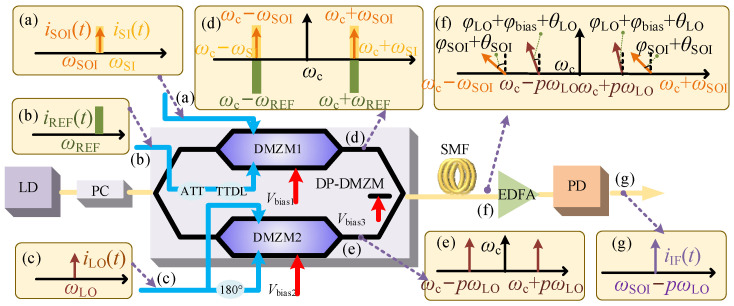
Schematic diagram of the proposed scheme with capabilities of SIC, high-efficiency HDC, and DIPF compensation. LD: laser diode; PC: polarization controller; DP-DMZM: dual-parallel dual-drive Mach–Zehnder modulator; DMZM: dual-drive Mach–Zehnder modulator; 180° HC: 180° hybrid coupler; ATT: attenuator; TTDL: tunable time delay line; SMF: single-mode fiber; EDFA: erbium- doped fiber amplifier; PD: photodetector.

Figure 1 shows the schematic diagram of the proposed scheme with capabilities of SIC, high-efficiency HDC, and DIPF compensation. The main device is a dual-parallel, dual-drive Mach–Zehnder modulator (DP-DMZM), which has two child dual-drive Mach–Zehnder modulators (DMZM_1_ and DMZM_2_) embedded in the two arms of the parent Mach–Zehnder interferometer (MZI). The optical carrier is generated from a laser diode (LD) before being sent to a polarization controller (PC) and injected into the modulator. After the PC, the polarization state of the optical carrier is aligned with the main axis of DP-DMZM. In the upper path of the modulator, the impaired received signal, which contains the weak SOI and high-power self-interference (SI) signal, is applied to one RF port of DMZM_1_; meanwhile, the reference (REF) signal is injected into the other RF port. The REF signal is pre-adjusted via an electrical attenuator (ATT) and tunable time delay line (TTDL) to match its amplitude and time delay with the SI signal. The DMZM_1_ is biased at the minimum transmission point (MITP) to achieve phase inversion between SI and REF signals, which enables the realization of SIC in the optical domain before fiber transmission. In the lower path of the modulator, the LO signal is applied to the two RF ports of DMZM_2_ through a 180° hybrid coupler (HC). By adjusting the DC bias of the sub-modulator, double sideband (DSB) signals with specific order can be generated to achieve HDC, which negates the requirement for a high-frequency LO signal source. Then, the output signal of DP-DMZM is transmitted over single-mode fiber (SMF). Due to the adjustable phase induced by the DC bias of the main modulator, the DIPF effect induced in optical fiber transmission can be compensated, accompanied by improved frequency conversion efficiency. Then, the power amplification and photoelectric conversion are realized through the erbium-doped fiber amplifier (EDFA) and photodetector (PD). Therefore, a pure intermediate frequency (IF) signal with improved frequency conversion efficiency and immunity to the DIPF effect is obtained, which is not disturbed by the SI signal.

Assume that the optical carrier is *E*_c_(*t*) = *E*_c_expj*ω*_c_*t*, where *E*_c_ and *ω*_c_ represent the amplitude and angular frequency of the optical carrier, respectively. *V*_LO_, *ω*_LO,_ and *φ*_LO_ represent the amplitude, angular frequency, and initial phase of the LO signal, respectively. The expressions of LO, SOI, SI, and REF signals are expressed as *V*_LO_sin(*ω*_LO_*t + φ*_LO_), *V*_SOI_sin(*ω*_SOI_*t + φ*_SOI_), *V*_SI_sin(*ω*_SI_*t + φ*_SI_), and *V*_REF_sin(*ω*_REF_*t + φ*_REF_). *V_i_*, *ω_i_,* and *φ_i_* represent the amplitude, angular frequency, and initial phase of LO, SOI, SI, and REF signals where *i* can be replaced by LO, SOI, SI, and REF. The optical modulated signals after DMZM_1_ and DMZM_2_ are expressed as follows, and the output spectra of sub-modulators are shown as insets (d) and (e) in Figure 1.

(1) $$E_{\mathrm{DMZ}M_{1}}(t)=\frac{E_{c}(t)}{10^{\left(IL/20\right)}}\times \left\{\gamma \exp\left\{\left[j\beta_{\mathrm{SOI}}\sin(\omega_{\mathrm{SOI}}t+\varphi_{\mathrm{SOI}})\right]+\left[j\beta_{\mathrm{SI}}\sin(\omega_{\mathrm{SI}}t+\varphi_{\mathrm{SI}})\right]\right\}+(1-\gamma )\exp\left[j\beta_{\mathrm{REF}}\sin(\omega_{\mathrm{REF}}t+\varphi_{\mathrm{REF}})\right]\times \exp(j\varphi_{\mathrm{bias}1})\right\}$$

(2) $$E_{\mathrm{DMZ}M_{2}}(t)=\frac{E_{c}(t)}{10^{\left(IL/20\right)}}\times \left\{\gamma \exp\left[j\beta_{\mathrm{LO}}\sin(\omega_{\mathrm{LO}}t+\varphi_{\mathrm{LO}})\right]+(1-\gamma )\exp\left[j\beta_{\mathrm{LO}}\sin(\omega_{\mathrm{LO}}t+\varphi_{\mathrm{LO}}+\pi )\right]\times \exp(j\varphi_{\mathrm{bias}2})\right\}$$

where *β*_SOI_*/β*_SI_*/β*_RI_*/β*_LO_ is the modulation index of SOI, SI, REF, and LO signals, which equals π*V_i_/V*_π_ (*i* can be replaced by SOI, SI, REF, and LO), respectively. *V*_π_ represents the half-wave voltage of the DP-DMZM. *φ*_bias1/2_ equals π*V*_bias1/2_*/V*_π_ representing the phase induced by DC biases of sub-modulators. *IL* is the parameter insertion loss, and *γ* denotes the power splitting ratio of both Y-branch waveguides (assumed to be symmetrical). *γ* is related to the extinction ratio of the modulator, which is given by

(3) $$\gamma =\left(1-\frac{1}{\sqrt{\varepsilon_{r}}}\right)/2\varepsilon_{r}=10^{ExtRatio/10}$$

where *ExtRatio* is linked to the parameter extinction ratio (ER) of the modulator.

This makes *φ*_bias1_ equal π to realize phase-inversion between SI and REF signal and expands Equation (1) based on Bessel functions. The sidebands higher than the first order have been ignored for simplified analysis, so Equation (1) is transferred into

(4) $$\begin{array}{cc} E_{\mathrm{DMZ}M_{1}}(t) & =\frac{E_{c}(t)}{10^{\left(IL/20\right)}}\times \left\{\gamma \sum_{n=-\infty }^{\infty }J_{n}(\beta_{\mathrm{SOI}})\exp\left[jn(\omega_{\mathrm{SOI}}t+\varphi_{\mathrm{SOI}})\right]\times \sum_{m=-\infty }^{\infty }J_{m}(\beta_{\mathrm{SI}})\exp\left[jm(\omega_{\mathrm{SI}}t+\varphi_{\mathrm{SI}})\right]-(1-\gamma )\sum_{k=-\infty }^{\infty }J_{k}(\beta_{\mathrm{REF}})\exp\left[jk(\omega_{\mathrm{REF}}t+\varphi_{\mathrm{REF}})\right]\right\}\\ & =\frac{E_{c}(t)}{10^{\left(IL/20\right)}}\times \left\{\begin{array}{l} \gamma J_{0}(\beta_{\mathrm{SOI}})J_{0}(\beta_{\mathrm{SI}})-(1-\gamma )J_{0}(\beta_{\mathrm{REF}})+\gamma J_{1}(\beta_{\mathrm{SOI}})J_{0}(\beta_{\mathrm{SI}})\left\{\begin{array}{l} \exp\left[j(\omega_{\mathrm{SOI}}t+\varphi_{\mathrm{SOI}})\right]-\\ \exp\left[-j(\omega_{\mathrm{SOI}}t+\varphi_{\mathrm{SOI}})\right] \end{array}\right\}\\ +\gamma J_{0}(\beta_{\mathrm{SOI}})J_{1}(\beta_{\mathrm{SI}})\left\{\begin{array}{l} \exp\left[j(\omega_{\mathrm{SI}}t+\varphi_{\mathrm{SI}})\right]-\\ \exp\left[-j(\omega_{\mathrm{SI}}t+\varphi_{\mathrm{SI}})\right] \end{array}\right\}-(1-\gamma )J_{1}(\beta_{\mathrm{REF}})\left\{\begin{array}{l} \exp\left[j(\omega_{\mathrm{REF}}t+\varphi_{\mathrm{REF}})\right]-\\ \exp\left[-j(\omega_{\mathrm{REF}}t+\varphi_{\mathrm{REF}})\right] \end{array}\right\} \end{array}\right\} \end{array}$$

where J*_n_*(*β*) represents the *n*th-order Bessel function of the first kind. To cancel the SI signal directly in the optical domain, the SI and REF-signal-modulated optical sidebands need to have the same arrival time and amplitude, indicating *γ*J_0_(*β*_SOI_)J_1_(*β*_SI_) = (1 − *γ*)J_1_(*β*_REF_) and *φ*_SI_ *= φ*_REF_. Since the SI signal is a time-delayed and power-attenuated copy of the known transmitted signal in the IBFD system, and the REF signal is also a copy extracted from the transmitted signal for SIC, frequency equality between SI and REF signal is naturally satisfied, namely, *ω*_SI_ *= ω*_REF_. In addition, it can be seen from Equation (3) that when the ER is ideal to infinity, the power splitting ratio of the Y-branch waveguides is close to 0.5, which is the optimal case. However, when the ER deteriorates, the power splitting ratio gradually deviates from 0.5, which has a certain influence on the optical outputs. Fortunately, the condition of amplitude matching can still be achieved by adjusting the ATT, showing a certain ability to cope with the deterioration of the extinction ratio. Therefore, by precisely tuning the amplitude and the arrival time of the REF signal by adjusting the ATT and TTDL, the SI signal can be completely cancellated before fiber transmission. The output of DMZM_1_ can be further expressed as

(5) $$E_{\mathrm{DMZ}M_{1}}(t)=\frac{E_{c}(t)}{\sqrt{2}}\times \left\{\gamma J_{0}(\beta_{\mathrm{SOI}})J_{0}(\beta_{\mathrm{SI}})-(1-\gamma )J_{0}(\beta_{\mathrm{REF}})+\gamma J_{1}(\beta_{\mathrm{SOI}})J_{0}(\beta_{\mathrm{SI}})\left\{\exp\left[j(\omega_{\mathrm{SOI}}t+\varphi_{\mathrm{SOI}})\right]-\exp\left[-j(\omega_{\mathrm{SOI}}t+\varphi_{\mathrm{SOI}})\right]\right\}\right\}$$

With respect to the lower path, by adjusting the DC bias of DMZM_2_, differently modulated sidebands will be obtained. When *φ*_bias2_ *=* π is satisfied, and the ER is ideal (making the power splitter ratio equal 0.5), the even sidebands and the optical carrier will be suppressed with odd sidebands reserved, which can achieve good carrier-suppressed double-sideband (CS-DSB) modulation. When the ER deteriorates, the optical carrier cannot be fully suppressed. There will be a residual optical carrier together with the optical carrier in the upper branch, which will cause LO and RF leakage. Fortunately, it can be filtered through a subsequent electrical filter. Therefore, the impact of ER deterioration on system performance is acceptable. Expanding Equation (2) based on Bessel functions under this condition, Equation (2) can be simplified as

(6) $$\begin{array}{cc} E_{\mathrm{DMZ}M_{2}}(t) & =\frac{E_{c}(t)}{10^{\left(IL/20\right)}}\times \left\{\left[\gamma -{\left(-1\right)}^{n}(1-\gamma )\right]\sum_{n=-\infty }^{\infty }J_{n}(\beta_{\mathrm{LO}})\exp\left[jn(\omega_{\mathrm{LO}}t+\varphi_{\mathrm{LO}})\right]\right\}\\ & =\frac{E_{c}(t)}{10^{\left(IL/20\right)}}\times \left[(2\gamma -1)J_{0}(\beta_{\mathrm{LO}})+\sum_{l=1}^{\infty }J_{2l-1}(\beta_{\mathrm{LO}})\left\{\exp\left[j(2l-1)(\omega_{\mathrm{LO}}t+\varphi_{\mathrm{LO}})\right]-\exp\left[-j(2l-1)(\omega_{\mathrm{LO}}t+\varphi_{\mathrm{LO}})\right]\right\}\right] \end{array}$$

In Equation (6), *l* is a positive integer. As shown in Equation (6), a (2*l*–1)-order carrier-suppressed double-sideband (CS-DSB) signal is obtained at the output of DMZM_2_, such as ±1st, ±3rd, and +5th-order CS-DSB signals. Then, the DSB signals with different order sidebands are combined with the reserved SOI-modulated signal at the output of DP-DMZM with the DC bias of the main modulator inducing a phase *φ*_bias3_ equaling π*V*_bias3_*/V*_π_. The optical signal at the output of DP-DMZM can be written as

(7) $$E_{\mathrm{DP-DMZ}M}(t)=\frac{E_{c}(t)}{10^{\left(IL/20\right)}}\times \left\{\begin{array}{l} \gamma J_{0}(\beta_{\mathrm{SOI}})J_{0}(\beta_{\mathrm{SI}})-(1-\gamma )J_{0}(\beta_{\mathrm{REF}})+\gamma J_{1}(\beta_{\mathrm{SOI}})J_{0}(\beta_{\mathrm{SI}})\left\{\exp\left[j(\omega_{\mathrm{SOI}}t+\varphi_{\mathrm{SOI}})\right]-\exp\left[-j(\omega_{\mathrm{SOI}}t+\varphi_{\mathrm{SOI}})\right]\right\}\\ +(2\gamma -1)J_{0}(\beta_{\mathrm{LO}})+\sum_{l=1}^{\infty }J_{2l-1}(\beta_{\mathrm{LO}})\left\{\exp\left[j(2l-1)(\omega_{\mathrm{LO}}t+\varphi_{\mathrm{LO}})\right]-\exp\left[-j(2l-1)(\omega_{\mathrm{LO}}t+\varphi_{\mathrm{LO}})\right]\right\}\times \exp(j\varphi_{\mathrm{bias}3}) \end{array}\right\}$$

Then, the signals output from DP-DMZM is transmitted over a single-mode fiber (SMF). The fiber dispersion will introduce a phase shift to the optically modulated signals through fiber transmission, making the transmitted signals experience a so-called power-fading effect. The optical signal after fiber transmission can be denoted as

(8) $$E_{\mathrm{SMF}}(t)=\frac{E_{c}(t)e^{-\alpha L/2}}{10^{\left(IL/20\right)}}\times \left\{\begin{array}{l} \gamma J_{0}(\beta_{\mathrm{SOI}})J_{0}(\beta_{\mathrm{SI}})-(1-\gamma )J_{0}(\beta_{\mathrm{REF}})+\gamma J_{1}(\beta_{\mathrm{SOI}})J_{0}(\beta_{\mathrm{SI}})\left\{\begin{array}{l} \exp\left[j(\omega_{\mathrm{SOI}}t+\varphi_{\mathrm{SOI}})\right]\\ -\exp\left[-j(\omega_{\mathrm{SOI}}t+\varphi_{\mathrm{SOI}})\right] \end{array}\right\}\times \exp\left(j\frac{\beta_{2}L\omega_{\mathrm{SOI}}{}^{2}}{2}\right)\\ +(2\gamma -1)J_{0}(\beta_{\mathrm{LO}})+\sum_{l=1}^{\infty }J_{2l-1}(\beta_{\mathrm{LO}})\left\{\begin{array}{l} \exp\left[j(2l-1)(\omega_{\mathrm{LO}}t+\varphi_{\mathrm{LO}})\right]-\\ \exp\left[-j(2l-1)(\omega_{\mathrm{LO}}t+\varphi_{\mathrm{LO}})\right] \end{array}\right\}\times \exp(j\varphi_{\mathrm{bias}3})\times \exp\left[j\frac{\beta_{2}L{\left(2l-1\right)}^{2}\omega_{\mathrm{LO}}{}^{2}}{2}\right] \end{array}\right\}$$

Here, *α*, *β*_2_, and L are the attenuation coefficient, second-order dispersion coefficient, and length of SMF. The output spectra of SMF are shown as inset (f) in Figure 1. Here, *θ*_SOI_ = exp(j*β*_2_*Lω*_SOI_^2^/2) and *θ*_LO_ = exp(j*β*_2_*Lω*_LO_^2^/2) represent the dispersion-induced phase shift of SOI and LO signals by optical fiber transmission. Then, the transmitted signals are sent to the EDFA to be amplified with a gain expressed as *G*_EDFA_. The amplified signal is injected into the PD, and the photocurrent after PD is expressed as

(9) $$i_{\mathrm{out}}(t)\propto {\left(-1\right)}^{m}\frac{\gamma A}{10^{\left(IL/10\right)}}\times \cos\left\{\frac{\beta_{2}L\left[\omega_{\mathrm{SOI}}{}^{2}-{\left(2l-1\right)}^{2}\omega_{\mathrm{LO}}{}^{2}\right]}{2}-\varphi_{\mathrm{bias}3}\right\}\times \left\{\begin{array}{l} \cos\left\{\left[\omega_{\mathrm{SOI}}-(2l-1)\omega_{\mathrm{LO}}\right]t+\varphi_{\mathrm{SOI}}-(2l-1)\varphi_{\mathrm{LO}}\right\}\\ -\cos\left\{\left[\left(\omega_{\mathrm{SOI}}+(2l-1)\omega_{\mathrm{LO}}\right)\right]t+\varphi_{\mathrm{SOI}}+(2l-1)\varphi_{\mathrm{LO}}\right\} \end{array}\right\}$$

where *A =* −4*e*^−*αL*^*RG*_EDFA_*E*_c_^2^J_1_*(β*_SOI_*)*J_0_*(β*_SI_*)*J_2*l* − 1_*(β*_LO_*)*, and *R* is the responsivity of the PD. As can be seen, the IF signal is generated accompanied by the frequency up-conversion signal, which can be removed by a subsequent low pass filter.

For a specific *φ*_bias3_*,* the power of the IF signal changes periodically with the fiber length and the frequencies of the SOI and LO signals. Especially, when *β*_2_*L*[*ω*_SOI_^2^ − (2*l* − 1)^2^*ω*_LO_^2^]/2 − *φ*_bias3_ = π/2 + *m*π is realized (*m* is integer), a serious power-fading effect occurs. Luckily, the phase difference *φ*_bias3_ can be tuned arbitrarily from 0 to 2π by adjusting the DC bias of the main modulator. Therefore, the optimal *φ*_bias3_ that satisfies *β*_2_*L*[*ω*_SOI_^2^ − (2*l* − 1)^2^ω_LO_^2^]/2 = *φ*_bias3_ + *m*π can be obtained to compensate for the power fading and maximize the RF signal, namely, cos{*β*_2_*L*[*ω*_SOI_^2^ − (2*l* − 1)^2^ω_LO_^2^]/2 − *φ*_bias3_} = (−1)*^m^*. After power compensation, the photocurrent of the frequency-converted IF signal is written as

(10) $$i_{\mathrm{out}}(t)\propto {\left(-1\right)}^{m}\frac{\gamma A}{10^{\left(IL/10\right)}}\left\{\cos\left[(\omega_{\mathrm{SOI}}-p\omega_{\mathrm{LO}})t+\varphi_{\mathrm{SOI}}-p\varphi_{\mathrm{LO}}\right]-\cos\left[(\omega_{\mathrm{SOI}}+p\omega_{\mathrm{LO}})t+\varphi_{\mathrm{SOI}}+p\varphi_{\mathrm{LO}}\right]\right\}$$

For the convenience of expression, 2*l* − 1 (*l* is a positive integer) is replaced by *p*. In the RF receiver with frequency down-conversion and self-interference cancellation, the power of the weak received RF signal is often unknown and relatively low. Therefore, the conversion gain deserves more attention to guide us to obtain a higher conversion efficiency and promote a better recovery of the received signals. The conversion gain of the receiver, defined as the power ratio between the IF and RF signals, is calculated as

(11) $$Gain\left(dB\right)=10\log\left(\frac{P_{\mathrm{IF}}}{P_{\mathrm{RF}}}\right)=10\log\left\{{\left[\frac{-4e^{-\alpha L}RG_{\mathrm{EDFA}}\gamma E_{c}{}^{2}\pi J_{0}\left(\beta_{\mathrm{SI}}\right)J_{p}\left(\beta_{\mathrm{LO}}\right)}{10^{\left(IL/10\right)}V_{\pi }}\right]}^{2}R_{\mathrm{out}}R_{\mathrm{in}}\right\}$$

*P*_IF_ and *P*_RF_ are the power of the IF and RF signals, respectively. *R*_out_ and *R*_in_ are the output- and input-matched impedance of the system. It can be seen from Equations (10) and (11) that the insertion loss of the modulator will cause a decline in the signal power and conversion gain. Fortunately, the insertion loss of common commercially available modulators is usually around 5 dB, and the loss can be compensated by optical amplifiers. In principle, the conversion gain can be improved by applying the PD with higher responsivity and modulator with smaller half-wave voltage, as well as setting a proper modulation index of the LO signal. Furthermore, since no self-interference exists in the DSB optical signal, fiber dispersion will not make the self-interference reappear after photodetection, which means it will be immune to fiber dispersion.

## 3. Simulation and Discussion

To validate the proposed approach, a simulation based on the setup shown in Figure 1 was carried out by using the commercial software “Optisystem 15.0” (Ottawa, ON, Canada). An optical carrier with a frequency of 193.1 THz and a power of 16 dBm was emitted from a laser dioxide (LD) and then sent to a polarization controller (PC) before being injected into the DP-DMZM. The PC was applied to adjust the polarization state of the optical carrier to align with one of the principal axes of the modulator. The half-wave voltage and extinction ratio of DP-DMZM were set as 3.5 V and 20 dB, respectively. The insertion loss of the modulator was set as a typical value of 5 dB. Parameters were set according to the actual parameters of the commercial modulator T.SBZH1.5-20PD-ADC. The upper radio frequency input port of the sub-modulator DMZM_1_ was driven by the impaired signals composed of the SOI and the SI signals, and the lower port was connected with the REF signal. A sinusoidal signal applied as the LO signal was first transmitted into a 180° hybrid to be divided into two paths with equal power and in inverse phase. The DMZM_1_ was biased at the MITP to achieve phase-inversion between SI and REF signals, and by adjusting the DC bias of the DMZM_2_, double sideband (DSB) signals with specific order were generated to achieve HDC. Then, the signals in the two paths were combined at the output of the main modulator. After 10 km single-mode fiber (SMF) transmission, which has an attenuation of 0.2 dB/km and a chromatic dispersion of 16.75 ps/nm·km, the output signals were amplified by an EDFA with a gain of 10 dB and sent into a PD with a responsivity of 0.95 A/W.

### 3.1. Verification of SIC and HDC Performance

**Figure 2:**
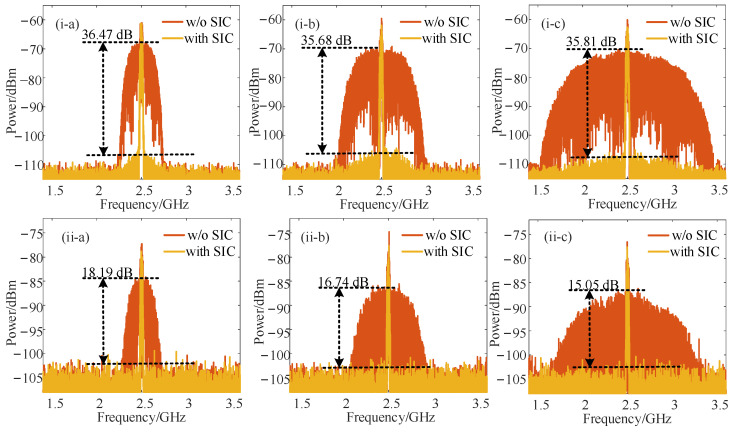
Electrical spectra of the down-converted IF signals with (yellow line) and without SIC (orange line) when the SOI signal was centered at (**i**) 12.5 GHz for fundamental down-conversion and (**ii**) 20.5 GHz for harmonic down-conversion with the SI bandwidth set as (**a**) 500 MHz, (**b**) 1 GHz, and (**c**) 2 GHz.

First, the SIC performance of the system was demonstrated. The LO signal was selected as a single-tone sinusoidal signal. The SOI signal was first set as a 16-quadrature amplitude modulation (QAM) modulated signal with a power of 10 dBm and a bandwidth of 50 MHz. The SI signal and REF signal were set as a 16-QAM modulated signal with a power of 20 dBm. The frequency of the SOI signal and SI signal were set to 12.5 GHz, and the corresponding LO signal frequency was 10 GHz. By beating with the +1st–order LO sideband and the +1st–order SOI (the interference signal at the same frequency) sideband in the PD, the down-conversion IF signal centered at 2.5 GHz was obtained. Simulation results of SIC performance with and without SIC are shown in Figure 2(i) when the bandwidth of the SI signal was set to 500 MHz, 1 GHz, and 2 GHz, successively. The output power of the LO signal was 13 dBm in this case. As can be seen from Figure 2(i), for SI signals with different bandwidths, satisfactory performance of SIC was achieved with a cancellation depth greater than 35 dB.

**Figure 3:**
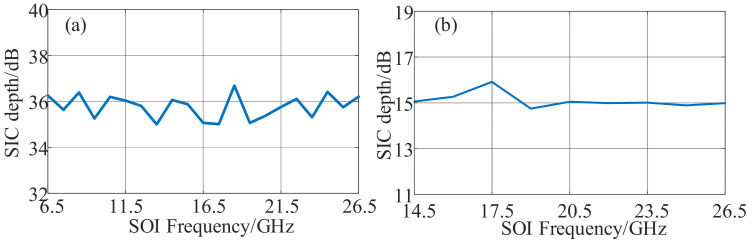
Measure curves of SIC depth versus SOI frequency with SIC enabled when (**a**) fundamental down-conversion and (**b**) harmonic down-conversion were achieved.

In order to demonstrate the HDC performance and the tunability of our scheme, we tuned the frequencies of the SOI/SI and LO signals to 20.5 GHz and 6 GHz. By beating the +3rd-order LO-modulated sideband and +1^st^-order SOI/SI signal sideband, the SIC and HDC were implemented successfully. The measured electrical spectra of IF signals with and without SIC when the bandwidth of the SI signal was set as 500 MHz, 1 GHz, and 2 GHz are shown in Figure 2(ii). The output power of the LO signal was increased to 27 dBm in order to obtain a high-power interference and down-converted IF signal. As can be seen, the SIC depth over 15 dB was successfully achieved with the SI signal suppressed under the noise. To study the self-interference cancellation performance under different received SOI frequencies, the carrier frequencies of the desired SOI signal and the self-interference signal were tuned from 6.5 to 26.5 GHz, and the frequency of the LO signal was correspondingly tuned from 4 to 24 GHz for fundamental down-conversion to ensure that the frequency of the IF signal remained at 2.5 GHz. The SI signal was a 16-QAM modulated signal with 2 GHz bandwidth, and other parameters were unchanged. Figure 3a shows the measured SIC depth curve of the received IF signal indicating that the SIC performance was satisfactory for different SOI frequencies with a SIC depth fluctuating around 36 dB. Then, the carrier frequencies of the desired SOI signal were tuned from 14.5 to 26.5 GHz, and the frequency of the LO signal was correspondingly tuned from 4 to 8 GHz for third-order harmonic frequency conversion. As shown in Figure 3b, the SIC depth fluctuated around 15 dB, showing that the SIC performance at different frequencies was basically the same.

Furthermore, the LO-modulated optical sideband with higher order was also available to down-convert signals in a higher-frequency band into an IF band. It should be noted that when *φ*_bias2_ = 0 was satisfied, the odd sidebands were suppressed with even sidebands reserved. Similarly, ±2st, ±4rd, and +6th-order DSB-modulated signals were obtained at the output of DMZM_2_, which are also usable for harmonic down-conversion. When the modulation index *β*_LO_ was further properly set up to 2.405, the *J*_0_(*β*_LO_) could approximately equal 0, indicating an optical carrier-suppressed condition was satisfied. Therefore, carrier-suppressed DSB-modulated signals with even sidebands were realized for harmonic down-conversion.

### 3.2. Recovery Performance of SOI

**Figure 4:**
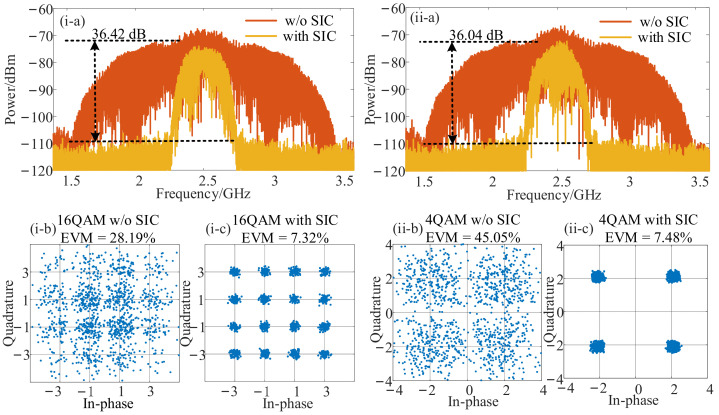
The SOI recovery performance for fundamental down-conversion when a 2 GHz SI signal and a 500 MHz SOI centered at 12.5 GHz with the modulation formats of (**i**) 16-QAM and (**ii**) 4-QAM were used. (**a**) The measured electrical spectra with and without SIC, the measured EVM (**b**) without and (**c**) with SIC.

Then, the recovery performance of the SOI signal was investigated. On the previous parameter setting, Figure 4(i) shows the SOI recovery performance when a 16-QAM SOI with 500 MHz bandwidth and a 2 GHz SI signal centered at 12.5 GHz were used. The output power of SOI and SI signals remained at 10 dBm and 20 dBm. The LO signal was centered at 10 GHz with a power of 13 dBm. As shown in Figure 4(i-a), the SOI was completely buried under the SI signal spectrum without SIC, and an unrecognizable constellation diagram shown in Figure 4(i-b) with an error vector magnitude (EVM) of 28.19% resulted. When the SIC was enabled, the wideband self-interference was canceled with a cancellation depth of 36.42 dB, leading to a completely visible SOI and a clear constellation diagram shown in Figure 4(i-c) with an EVM of 7.32%. The down-converted IF signal was demodulated and recovered through offline digital signal processing (DSP), which included normalization, equalization, and EVM calculation. To investigate the SOI recovery performance for various modulation formats, a 4-QAM signal with the same bandwidth and frequency was applied as the SOI, whereas the parameters of the SI signal remained unchanged. The spectrum of IF signals and demodulated constellation with and without SIC is shown in Figure 4(ii). When SIC was disabled, the SOI was disturbed by the wideband SI signal, and a 36.04 dB cancellation depth was achieved when the SIC was enabled, as shown in Figure 4(ii-a). The SI signal suppressed the noise, and the IF signal was successfully recovered. Therefore, as shown in Figure 4(ii-b,ii-c), the EVM was reduced from 45.05% to 7.48%, indicating a satisfactory recovery performance.

**Figure 5:**
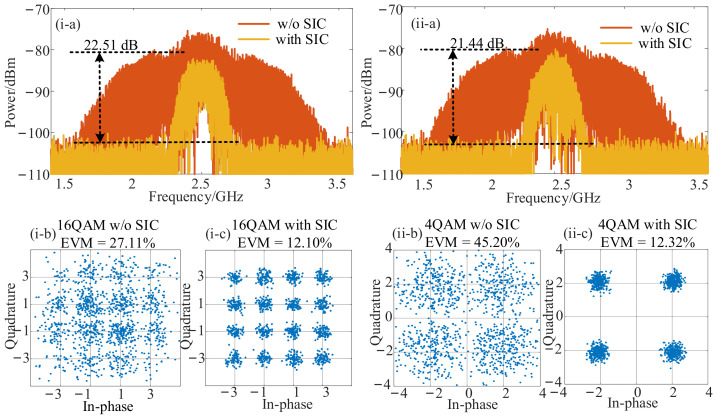
The SOI recovery performance for harmonic down-conversion when a 2 GHz SI signal and a 500 MHz SOI centered at 20.5 GHz with the modulation formats of (**i**) 16-QAM and (**ii**) 4-QAM were used. (**a**) The measured electrical spectra with and without SIC, the measured EVM (**b**) without and (**c**) with SIC.

In order to investigate the recovery performance when HDC was performed, the frequencies of the SOI/SI and LO signals were switched to 20.5 GHz and 6 GHz. The LO signal power was increased to 30 dBm to obtain more intuitive and clear results. Other parameters were consistent with the previous analyses. As shown in Figure 5(i), when the SIC structure was used, the SIC cancellation depth reached 22.51 dB (Figure 5(i-a)), and the demodulated EVM was reduced from 27.11% (Figure 5(i-b)) to 12.10% (Figure 5(i-c)) which still complied with the 3GPP-specified EVM requirements for the 16-QAM signal below 12.5%. Then, the modulation format of SOI was changed to 4-QAM, and the self-interference cancellation and recovery results were obtained in the same way. Therefore, the wideband SIC depth was 21.44 dB, as shown in Figure 5(ii-a), and the 4-QAM SOI was recovered with an EVM value of 12.32%, as demonstrated in Figure 5(ii-c).

**Figure 6:**
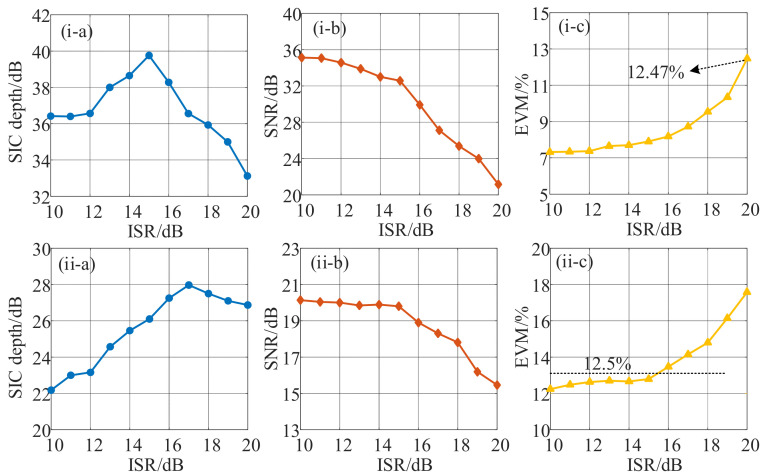
Measurement curves of (**a**) SIC depth, (**b**) SNR, and (**c**) EVM of received IF signal versus ISR with SIC enabled when the SOI was a 16 QAM modulated signal with a bandwidth of 500 MHz centered at (**i**) 12.5 GHz for fundamental down-conversion and (**ii**) 20.5 GHz for harmonic down-conversion.

The cancellation and recovery performance of SOI with different interference-to-signal ratios (ISRs) was also investigated, corresponding to the previous analyses of 16-QAM modulation with fundamental down-conversion and harmonic down-conversion. The power of the SOI signal remained unchanged at 10 dBm, while the power of the SI signal switched with a step of 1 dBm from 20 dBm to 30 dBm, corresponding to the ISR change from 10 dB to 20 dB. The other parameters were unchanged. Figure 6 shows the measurement curves of the SIC depth, the signal-to-noise ratio (SNR), and EVM. As can be seen in Figure 6(i), for the fundamental down-conversion, with the increase in ISR, the SIC depth (Figure 6(i-a)) first increased and then decreased, while the SNR (Figure 6(i-b)) generally deteriorated. The changing trend of SIC depth is due to the power of the interference signal being increased initially, and the SI signal was basically canceled under the noise, which leads to an increase in SIC depth. While with the continuous increase in SI signal power, the cancellation performance deteriorated, resulting in a decline in SIC depth. As for SNR, with the increase in the SI signal power, the value of J_0_(*β*_SI_) was reduced, so the power of the IF signal dropped slowly. As the power of the SI signal continued to increase when the ISR exceeded 15 dB, the SIC performance deteriorated, leading to a sharp decline in the SNR. Fortunately, a demodulated EVM (Figure 6(i-c)) satisfying the 3GPP-specified EVM limit for the 16 QAM signal could be achieved when the ISR was below 20 dB. As for harmonic down-conversion, the principle of SIC depth (Figure 6(ii-a)), SNR (Figure 6(ii-b)), and EVM (Figure 6(ii-c)) change trends are consistent with the analysis of fundamental down-conversion. A satisfactory EVM below 12.5% can be achieved when the ISR does not exceed 15 dB.

### 3.3. High-Efficiency Frequency Conversion and Compensation of DIPF

**Figure 7:**
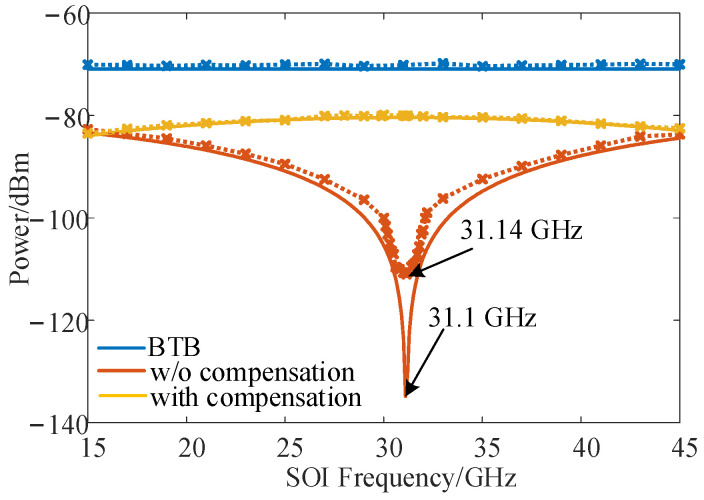
Measured IF power as a function of the SOI frequency in the theoretical analysis (solid line) and in the simulation (dashed line).

**Figure 8:**
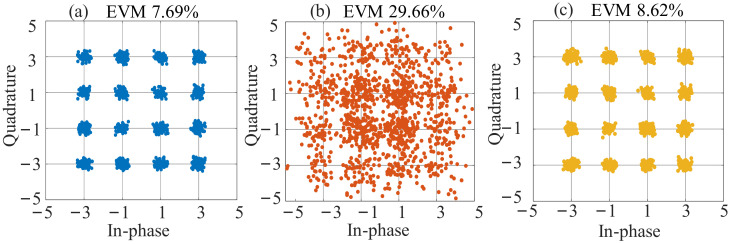
Demodulated constellation diagrams of IF signal when the SOI signal was centered at 31.1 GHz (**a**) without transmission over fiber (**b**), without DIPF compensation, and (**c**) with DIPF compensation over 25 km fiber transmission with SIC enabled.

For an RoF link, the ability to overcome the dispersion-induced power-fading effect is important for the improvement in SOI recovery, so the DIPF compensation performance for long-distance fiber transmission was demonstrated. The fiber length was set as 25 km in the simulated long-distance optical fiber transmission scenario. Taking fundamental down-conversion as an example, the theoretical curve of the IF signal power versus the SOI frequency was drawn for the conventional DSB-modulated frequency down-conversion link without DIPF compensation. The frequency of the SOI and SI signals was tuned from 15 GHz to 45 GHz, and the LO signal was tuned from 12.5 GHz to 42.5 GHz with a fixed IF signal centered at 2.5 GHz. In Figure 7, the solid orange line shows a power fading point near 31.1 GHz. According to Equation (8), a proper DC bias of the main modulator was calculated for power compensation in this case, and as the solid yellow line in Figure 7 shows, the fading point in the solid orange line switched to peak points in the solid yellow line. As a reference, the curve without transmission over fiber (back to back, BTB) was plotted in a solid blue line when the main DC-bias-induced phase shift was zero. Next, based on the simulation setup, a 16-QAM SOI with 500 MHz bandwidth and a 2 GHz SI signal were used in the following simulation. The output power of SOI and SI signals remained at 10 dBm and 20 dBm. The LO signal power was 13 dBm. The detected IF power as a function of the SOI frequency is also plotted in Figure 7 for the case when the SIC is enabled. The fading point is at 31.14 GHz, which basically agrees with the theoretical results, as shown by the dashed orange line. In addition, the simulation results were consistent with the theoretical analysis, showing good performance for DIPF compensation, as shown by the dashed yellow line. The demodulated constellation diagram of the IF signal when the SOI signal is centered at 31.1 GHz with SIC enabled is also shown in Figure 8. Without fiber transmission, the demodulated constellation diagram is clear, with a measured EVM of 7.69%. After 25 km of fiber transmission and without compensation, the SOI frequency was at the power fading point, and the EVM deteriorated from 7.69% to 29.66%, which made effective communication completely impossible. Fortunately, after adjusting the DC bias of the main modulator for DIPF compensation, the IF power was compensated well, and the EVM recovered to 8.62%.

The conversion gain also deserves to be discussed to obtain a higher conversion efficiency and promote better recovery of the received signals. In Ref. [23], a system to simultaneously realize SIC, CDIP elimination, and HDC was proposed by virtue of harmonic odd-order SSB down-conversion. The SSB-modulated link was immune to dispersion, while compared with the DSB-modulated link, the RF modulation efficiency was low and signal power was reduced. For power-insensitive links, the loss of signal power and frequency conversion gain caused by SSB modulation can be negligible. However, in a self-interference cancellation system with weak received signals, the improvement in conversion efficiency promotes signal recovery performance. In the SSB modulation-based scheme of Ref. [23], the optical power of the SOI-modulated sideband was reduced by 3 dB compared with the DSB-modulated scheme. Therefore, the gain ratio between our proposed scheme and the scheme in Ref. [23] is 6 dB, indicating a conversion gain improvement of 6 dB, which is conducive to weak signal recovery. In the scenarios with known received SOI frequency, the DC bias can be set in advance for compensation, and there is no need to perform other operations for a fixed SOI frequency in a settled transmission link.

Table 1 provides a performance comparison between this work and other reported methods in terms of functions, main components, cancellation bandwidth, cancellation depth, and fiber transmission capability. To date, photonic-assisted RF SIC schemes have shown wide bandwidth with a large cancellation depth [12,13,14,16], a combination of frequency conversion functions [15,17,18,19,20], or long-distance fiber transmission [13,14,15,16,17,18,20]. Nevertheless, compared with the proposed scheme, the scheme in Refs. [12,13,14] is not multifunctional, and the scheme in Refs. [15,17,18,19,20] is based on fundamental frequency tones, which cannot alleviate the demand for high-frequency LO sources. In addition, compared with the SSB modulation-based scheme in Ref. [23], the DSB modulation of the proposed scheme helps improve the frequency conversion efficiency, which is beneficial to signal recovery. Therefore, the proposed scheme can perform well for simultaneous ultrawideband SIC, high-speed SOI recovery, and fiber dispersion immunity with the capability of harmonic down-conversion and improved conversion efficiency.

## 4. Conclusions

In summary, a photonic-assisted scheme for simultaneous wideband SIC, high-speed SOI recovery, and HDC was proposed in theory and verified by simulations. The scheme was mainly based on a commercial DP-DMZM and showed a compact configuration and high cost-effectiveness. Thanks to self-interference cancellation in the optical domain, a phase control mechanism induced by fiber dispersion, and accurate DC bias regulation, the cancellation performance was immune to fiber transmission, and the DIPF effect was compensated with an improved conversion efficiency. The simulation results showed that a 500 MHz modulated SOI in X and K bands was down-converted to 2.5 GHz through fundamental and harmonic frequency conversion, respectively. The wideband self-interference was canceled over 35 dB and 20 dB after 10 km fiber transmission, and the SOI was recovered with an EVM, satisfying the standard of the 3GPP-specified EVM limit. The performance of signal recovery and SIC versus different ISR was also investigated. Furthermore, the simulation results confirm that DIPF compensation was achieved, and a 6 dB improvement in conversion efficiency was obtained compared with previous work, which contributes to effective signal recovery.

## Figures and Tables

**Table 1:** Comparison of various schemes.

Scheme	Functions	Main Components	Center Frequency (GHz)	Bandwidth (GHz)	SIC (dB)	Fiber Transmission (km)
This work	SIC, HDC, and DIPF compensation	DP-DMZM	2.5	2	36	10
[12]	SIC	Two parallel MZMs	18	10	30	/
[13]	SIC and anti-dispersion transmission	DP-QPSK	12	2	26	37
[14]	SIC and anti-dispersion transmission	PDM-MZM	15	2	17.6	10
[15]	Frequency down-conversion, SIC, and DIPF compensation	Dpol-MZM	1/3/5/7	0.04	28	25
[16]	SIC and DIPF compensation	Two parallel DPMZMs	2.5	2.7	30	25
[17]	SIC, frequency down-conversion, anti-dispersion transmission	DP-BPSK	1	0.1	23.7	25
[18]	SIC and image rejection mixing	Two parallel DPMZMs	2.5	1	21	25
[19]	SIC, LO generator, and frequency down-conversion	IQ-MZM	2.6	0.3	27.2	/
[20]	SIC, LO generator, frequency down-conversion, and DIPF compensation	DP-BPSK	2	1	21.2	35
[23]	SIC, HDC, and anti-dispersion transmission	DP-DDMZM	1	0.2	22	/

## Data Availability

Not applicable.
